# Opening of the Session of the Buffalo Medical College

**Published:** 1858-11

**Authors:** 


*Opening of the Session of the Buffalo Medical College.*—The present session will open with unusually encouraging indications of a good class. There are now, while we write, between twenty-five and thirty students in town, and by the time the Journal is issued there will probably be many more. From some of the accounts we have received from private sources, we hear that the sessions of the larger schools will also open with large classes. There is no doubt but that there are now more students in the country than there have been for some years: the commercial crisis has undoubtedly led more young men to turn their attention to the profession. With this general increase in the number of medical students, the undoubted claims of the Buffalo school upon the confidence of the profession, will insure for it a good class. In no place, with the exception of the larger cities, are the advantages of clinical instruction which are at the command of students, surpassed; and we conceive that the energy of the faculty in this respect places the real benefit to be derived from the actual observation of disease, on a par with that enjoyed in any city. We now have the advantages of two hospitals, with both of which, a portion of the faculty are connected. One of these, the Buffalo Hospital of the Sisters of Charity, is directly behind the college edifice, and the other is but a short distance removed. Those students who have desired it have always had at least one opportunity of seeing cases of variola at the pest house, where there are always a few cases. Every available means of instruction, then, are made use of to their fullest extent. Those students who have been here during the preliminary term, have been actively engaged in the prosecution of practical anatomy under the direction of the demonstrator, and abundant facilities will be afforded for the study of this branch throughout the term. In addition to the regular course of instruction, lectures will be given by the editor of this Journal, on physiology, illustrated by experiments upon living animals after the method first inaugurated in this country, *at this institution*, by Prof. Dalton, now of the College of Physicians and Surgeons at New York. Buffalo has reason to be proud of her medical school, which has been steadfast to its principles, in the days when medical colleges, with few exceptions, were not very flourishing. That time is passing away, and we predict a large class for this year, and a larger one for the next. Those whom this notice finds undecided where to go, cannot do better than to come to Buffalo. The regular term will be opened on the first Wednesday in November, by an introductory lecture by Dr. Mack, the lecturer on Materia Medica and Therapeutics.

